# ROS Production and ERK Activity Are Involved in the Effects of d-β-Hydroxybutyrate and Metformin in a Glucose Deficient Condition

**DOI:** 10.3390/ijms18030674

**Published:** 2017-03-21

**Authors:** Santosh Lamichhane, Tonking Bastola, Ramesh Pariyar, Eun-Sol Lee, Ho-Sub Lee, Dae Ho Lee, Jungwon Seo

**Affiliations:** 1 Institute of Pharmaceutical Research and Development, College of Pharmacy, Wonkwang University, Iksan 570-749, Korea; lakki38786@gmail.com (S.L.); tonkingbastola@gmail.com (T.B.); ume.ramesh@gmail.com (R.P.); Chori0509@naver.com (E.-S.L.); 2 Hanbang Body-Fluid Research Center, Wonkwang University, Iksan 570-749, Korea; host@wku.ac.kr; 3 College of Oriental Medicine, Wonkwang University, Iksan 570-749, Korea; 4 Department of Internal Medicine, Gachon University Gil Medical Center, Incheon 21565, Korea

**Keywords:** hypoglycemia, d-β-hydroxybutyrate, metformin, reactive oxygen species, extracellular signal-regulated kinase

## Abstract

Hypoglycemia, a complication of insulin or sulfonylurea therapy in diabetic patients, leads to brain damage. Furthermore, glucose replenishment following hypoglycemic coma induces neuronal cell death. In this study, we investigated the molecular mechanism underlying glucose deficiency-induced cytotoxicity and the protective effect of d-β-hydroxybutyrate (D-BHB) using SH-SY5Y cells. The cytotoxic mechanism of metformin under glucose deficiency was also examined. Cell viability under 1 mM glucose (glucose deficiency) was significantly decreased which was accompanied by increased production of reactive oxygen species (ROS) and decreased phosphorylation of extracellular signal-regulated kinase (ERK) and glycogen synthase 3 (GSK3β). ROS inhibitor reversed the glucose deficiency-induced cytotoxicity and restored the reduced phosphorylation of ERK and GSK3β. While metformin did not alter cell viability in normal glucose media, it further increased cell death and ROS production under glucose deficiency. However, D-BHB reversed cytotoxicity, ROS production, and the decrease in phosphorylation of ERK and GSK3β induced by the glucose deficiency. ERK inhibitor reversed the D-BHB-induced increase in cell viability under glucose deficiency, whereas GSK3β inhibitor did not restore glucose deficiency-induced cytotoxicity. Finally, the protective effect of D-BHB against glucose deficiency was confirmed in primary neuronal cells. We demonstrate that glucose deficiency-induced cytotoxicity is mediated by ERK inhibition through ROS production, which is attenuated by D-BHB and intensified by metformin.

## 1. Introduction

Glucose is the key source of energy necessary for brain functioning, and hypoglycemia has damaging effects on various organs, including the brain [1]. Hypoglycemia can arise because of starvation, disturbance in growth hormone production, and development of tumors from insulin producing pancreatic islet cells, and it can also be caused by injection of insulin in an amount above the therapeutic dose [2]. In most people with type 1 diabetes mellitus and in many people with advanced type 2 diabetes mellitus, acute severe hypoglycemia induced by insulin or sulfonylurea therapy is one of the main complications that induce brain damage. A decrease in the glucose inflow to the brain can cause convulsions, coma [2], and even death. According to electroencephalography data, a decrease in the blood glucose level below 2 mM is associated with a decrease in brain activity [2]. A decrease in the glucose level below 1 mM results in ATP depletion in the brain tissue and causes irreversible damage to the central nervous tissue [2]. Glucose deprivation has been reported to result in ATP depletion and oxidative stress in a tight association with mitochondrial dysfunction [3]. However, the underlying molecular mechanism has not been fully understood. In particular, low blood glucose effect mimicking hypoglycemia remains to be elucidated, especially in neuronal cells and brain.

Furthermore, it has been reported that hyperglycemia following hypoglycemic coma can induce neuronal cell death by the mechanism associated with extracellular zinc release and activation of nicotinamide adenine dinucleotide phosphate (NADPH) oxidase [4]. Therefore, further studies for identifying safe and effective treatments for hypoglycemic coma are needed to prevent glucose-reperfusion injury. Ketone bodies, acetoacetate, and d-β-hydroxybutyrate (D-BHB) are known to be the alternative energy sources of glucose in the mammalian brain, and they become an important substrate for energy production in the brain during prolonged starvation [5]. Besides providing energy to neurons, ketone bodies decrease the production of reactive oxygen species (ROS) induced by glutamate treatment [6,7] or glycolysis inhibition [8] in cultured neurons. The neuroprotective effects of ketone bodies in vivo have been reported in various pathological conditions including ischemia [9,10] and hypoglycemia [8,11]. In the hippocampus of a rat model of insulin-induced hypoglycemia, administration of D-BHB, and not acetoacetate, reversed hypoglycemia-induced lipid peroxidation [8]. In addition, D-BHB administration decreased ROS levels and reversed neuronal cell death in the cortex of hypoglycemic rats [12]. Therefore, D-BHB might be a potential therapeutic option for the treatment of hypoglycemia-induced brain damage.

Metformin (*N*′,*N*′-dimethylbiguanide) is one of the most widely prescribed drugs for T2DM patients [13]. Metformin therapy has been reported to reduce cancer incidence and mortality in diabetic patients [14,15], and it has some cardiovascular benefits [16]. Metformin inhibits tumor growth in xenograft models of various cancers [17,18,19]. In a xenograft mouse model of neuroblastoma, metformin inhibited tumor growth through the activation of Rac1 and CDC42 and inactivation of RhoA [20]. In SH-SY5Y cells, metformin effectively suppressed cell proliferation, invasion potential, and sphere-forming ability [21,22]. On the contrary, metformin attenuated the cytotoxicity induced by 1-methyl-4-phenylpyridinium (MPP^+^) [23] or α-synuclein [24] in SH-SY5Y cells. Interestingly, it was reported that lowering the glucose level potentiated metformin-induced cytotoxicity in breast cancer cells [25]. In this regard, the glucose level might account for this discrepancy between the anti-cancer effect and neuroprotection in SH-SY5Y cells.

In this study, we investigated the molecular mechanism underlying glucose deficiency-induced cell death in SH-SY5Y cells. Furthermore, we examined the effects of metformin and D-BHB in a glucose deficient condition and elucidated their underlying molecular mechanisms. We demonstrate that glucose deficiency-induced cell death is mediated by ROS production, which is attenuated by treatment with ketone body D-BHB. In addition, metformin has a cytotoxic effect only in a glucose deprivation condition, which is also mediated by ROS production.

## 2. Results

### 2.1. Glucose Deficiency Decreases Cell Viability and Intracellular ATP Level in SH-SY5Y Cells

**Figure 1:**
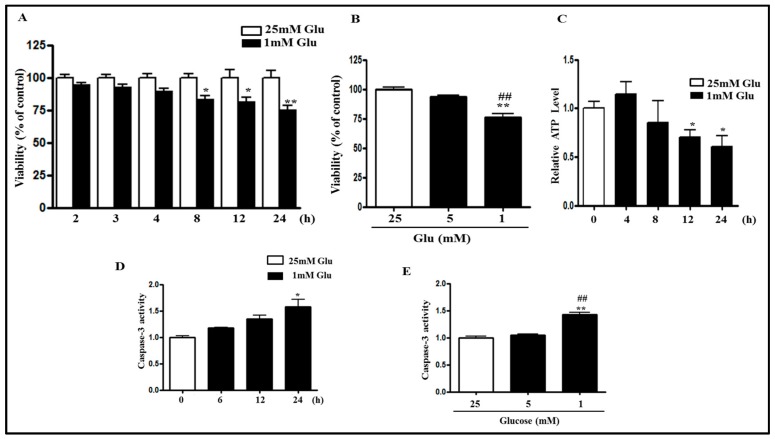

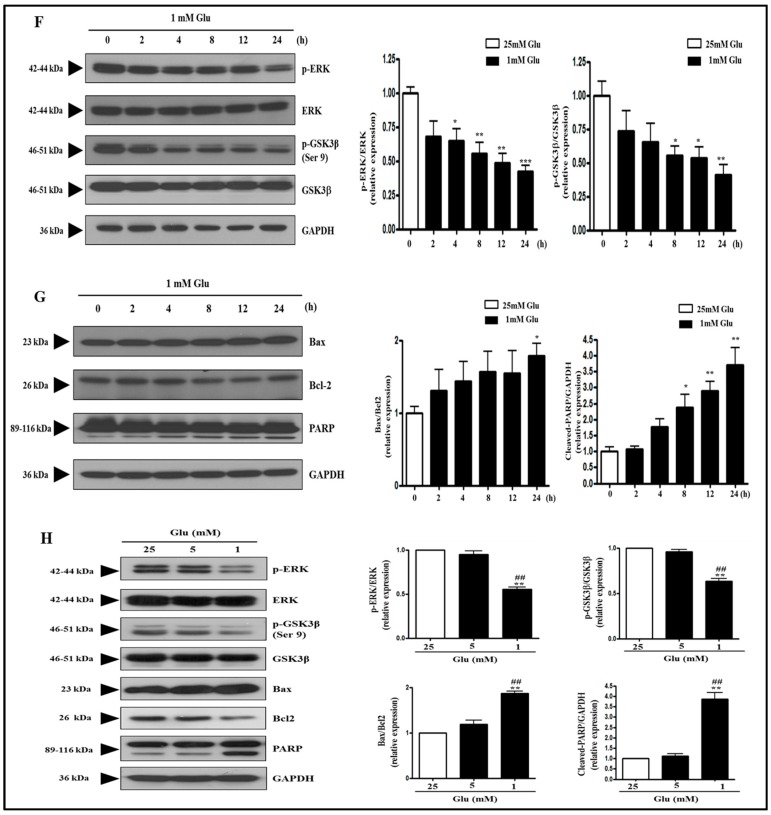
Glucose deficiency decreases cell viability, intracellular ATP levels, and the phosphorylation of ERK and GSK3β in SH-SY5Y cells. SH-SY5Y cells were treated with (**A**) the media containing 25 mM or 1 mM glucose for the indicated time or with (**B**) 25, 5, or 1 mM glucose for 24 h. The cell viability was then measured using 3-(4,5-Dimethylthiazol-2-yl)-2,5-Diphenyltetrazolium Bromide (MTT) assay. Each bar represents the mean percentage alteration above or below the control (±S.D.) (*n =* 4–6); (**C**) The cells were treated with 1 mM glucose for 4, 8, 12, or 24 h. Control cells (0 h) were treated with 25 mM glucose. Intracellular ATP levels were detected by ATP measurement assay as described in the “Materials and Methods” section. Each bar graph shows the mean fold alteration above or below the control (±S.D.) (*n =* 3); (**D**–**E**) The cells were treated with (**D**) 25 or 1 mM glucose for the indicated time or with (**E**) 25 or 5 or 1 mM for 24 h. Caspase-3 activity was measured using caspase-3 activity assay kit as described in the ‘Materials and Methods’ section. (**F**) The protein levels of p-ERK, ERK, p-GSK3β, GSK3β, and glyceraldehyde 3-phosphate dehydrogenase (GAPDH) were measured in 1 mM glucose treated cells for the indicated time by Western blot analysis using specific antibodies; (**G**) The protein levels of Bax, Bcl-2, poly ADP ribose polymerase (PARP), and GAPDH were measured by Western blot analysis; (**H**) The protein levels of p-ERK, ERK, p-GSK3β, GSK3β, Bax, Bcl-2, PARP, and GAPDH were measured in 25, 5, or 1 mM glucose treated cells by Western blot analysis. The representative blots are shown and densitometry was used to quantify the protein levels. Each bar graph shows the mean fold decrease below the control (±S.D.) (*n =* 3). Differences were statistically significant at * *p* < 0.05, ** *p* < 0.01, and *** *p* < 0.001 as compared with 25 mM Glu and ## *p* < 0.01 as compared with 5 mM Glu.

Previous studies have mentioned that glucose deprivation induced apoptotic cell death in PC12 cells [3]. Therefore, we first investigated the viability of SH-SY5Y cells during a glucose deficient condition. Cell viability in the media containing 1 mM glucose was compared with that in the media containing 25 mM glucose, which is generally used for maintenance of SH-SY5Y cells. Methylthiazolyldiphenyl-tetrazolium bromide (MTT) assay showed that as time passed, cell viability was decreased from 2 h onwards, and it was significantly decreased at 8 h, and was further decreased at 24 h (Figure 1A). Although the media containing 25 mM glucose are usually used for general cell culture, the normal fasting blood glucose level for humans is from 3.9 to 5.5 mM. To evaluate the effect of the normal blood glucose concentration, the cell viability assay was performed in SH-SY5Y cells cultured with 25, 5, or 1 mM glucose containing media for 24 h. The cell viability assay showed that there is no significant difference in the cell viability between 25 mM glucose containing media and 5 mM glucose containing media (Figure 1B), meanwhile there was less than a 5% loss in the viability of the cells incubated with 5 mM glucose as compared to that with 25 mM glucose. The cell viability incubated with 1 mM glucose was significantly decreased as compared to that with 5 mM glucose as well as 25 mM glucose. This data confirmed that glucose deficiency decreases the cell viability and that 25 mM glucose condition used for general cell culture is not different from 5 mM glucose condition of normal human blood levels, at least in the aspect of cell viability. Although ATP and other intermediates of the biosynthetic process are generated from glucose metabolism, the intracellular ATP level was reported to be increased 3 h after glucose deprivation [3]. Consistent with that report, ATP content was slightly increased for up to 4 h of glucose deficiency, but it was significantly decreased from 12 h onwards (Figure 1C). However, the ATP level was more than 50% even at 24 h after glucose deficiency. Next, we examined if the decrease of cell viability is attributable to apoptosis induction using caspase-3 activity assay. Glucose deficiency increased caspase-3 activity in a time-dependent manner (Figure 1D) and caspase-3 activity in 1 mM glucose condition was increased as compared with 5 or 25 mM glucose, while there was no significant difference in caspase-3 activity between 5 and 25 mM glucose (Figure 1E).

It has been demonstrated that extracellular signal-regulated kinase (ERK)/glycogen synthase 3 (GSK3β) has a diverse role in the regulation of apoptosis [26,27,28] and cell survival [29,30]. Therefore, we detected the phosphorylation of ERK at Thr 202/Tyr 204 (ERK1) and Thr 185/Tyr 187 (ERK2) sites and the phosphorylation of GSK3β at Ser 9 site using Western blot analysis. Glucose deficiency decreased the phosphorylation of ERK and GSK3β (Figure 1F,H), suggesting the involvement of ERK and GSK3β in glucose deficiency-induced cell death. The pro-apoptotic enzyme Bax and the anti-apoptotic enzyme Bcl-2 play a crucial role in regulating cell death and survival [2,31]. Western blot analysis demonstrated that the expression of Bcl-2 protein was decreased, whereas the expression of Bax protein was slightly increased as compared to the control in a glucose deficient condition (Figure 1G,H). Poly (ADP-ribose) polymerase (PARP) is cleaved by caspase-3 activation, which is implicated in apoptotic cell death [32]. PARP cleavage was also progressively increased in glucose deficient condition, suggesting the induction of apoptosis (Figure 1G,H).

### 2.2. Glucose Deficiency Induces Cell Death through ROS Accumulation in SH-SY5Y Cells

**Figure 2:**
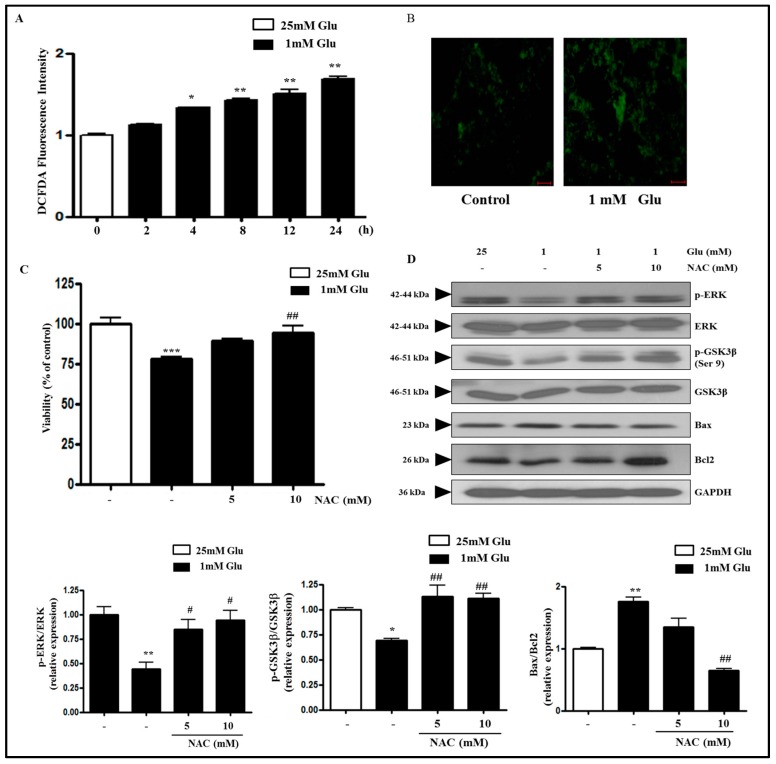
Glucose deficiency induces ROS production and *N*-acetyl-l-cysteine (NAC) reverses cytotoxicity and restores the decreased phosphorylation of ERK and GSK3β induced by glucose deficiency in SH-SY5Y cells. (**A**) The cells were treated with 1 mM glucose for 2, 4, 8, 12, or 24 h. Control cells (0 h) were treated with 25 mM glucose. ROS levels were measured by 2-7-Dichlorodihydrofluorescein diacetate (DCFDA) fluorescence assay. Each bar graph represents the mean fold increase above the control (±S.D.) (*n* = 3). Differences were statistically significant at * *p* < 0.05 and ** *p* < 0.01; (**B**) The cells were stained with DCFDA and the images were taken using a fluorescence microscope. The representative images are shown (scale bar = 50 μm); (**C**) The cells were treated with ROS inhibitor NAC (5 or 10 mM) in the media containing 1 mM glucose for 24 h. Control cells were treated with 25 mM glucose. The cell viability was measured by MTT assay. Results are expressed as the mean percentage alteration above or below the control (±S.D.) (*n =* 4–6); (**D**) The protein levels of p-ERK, ERK, p-GSK3β, GSK3β, Bax, Bcl2, and GAPDH were measured by Western blot analysis. The representative blots are shown and densitometry was used to quantify the protein levels. Each bar graph shows the mean fold alteration above or below the control (±S.D.) (*n* = 3). Differences were considered statistically significant at ** *p* < 0.01 and *** *p* < 0.001 vs. 25 mM Glu and # *p* < 0.5 and ## *p* < 0.01 vs. 1 mM Glu.

It has recently been shown that hypoglycemia induced oxidative stress in nerve tissue [33] and increased the production of ROS in cultured neurons and cells of neuronal origin [2,3,34]. Therefore, we tested the possibility of ROS accumulation during a glucose deficient condition in SH-SY5Y cells using 2-7-Dichlorodihydrofluorescein diacetate (DCFDA) fluorescence assay. The level of ROS was significantly and progressively increased after 4 h compared with the control (Figure 2A,B). Because MTT assay showed that glucose deficiency induced cytotoxicity (Figure 1A), we next investigated whether the cytotoxicity was attributable to the production of ROS. Glucose deficiency-decreased viability was significantly restored by co-treatment with ROS inhibitor *N*-acetyl-l-cysteine (NAC) (Figure 2C), suggesting that cell death induced by glucose deficiency is mediated by increased ROS production. In addition, NAC treatment restored the phosphorylation of ERK and GSK3β, which was decreased by 1 mM glucose treatment, while it attenuated the Bax/Bcl2 ratio increased by 1 mM glucose treatment (Figure 2D). These data indicate that glucose deficiency decreases the phosphorylation of ERK and GSK3β and increases apoptotic Bax/Bcl2 ratio through the increased ROS production.

### 2.3. Metformin Aggravates Glucose Deficiency-Induced Cell Death in SH-SY5Y Cells

**Figure 3:**
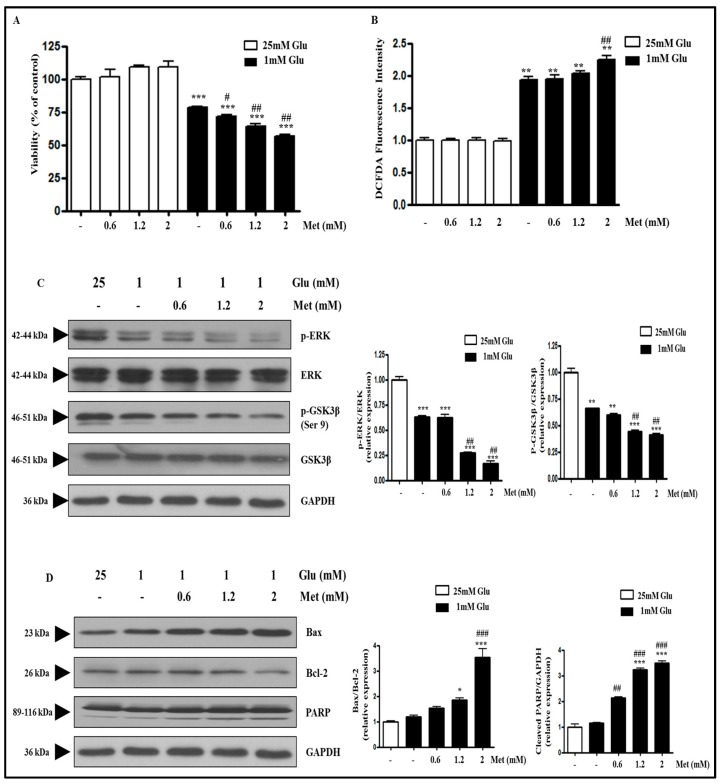
Metformin treatment further decreases cell viability and increases ROS production in glucose-deprived SH-SY5Y cells. SH-SY5Y cells were pre-incubated with the media containing 25 or 1 mM glucose for 4 h and then they were treated with metformin (0.6, 1.2, or 2 mM) for 24 h. (**A**) The cell viability was measured by MTT assay. Results are expressed as the mean percentage alteration above or below the control (25 mM Glu.) (±S.D.) (*n =* 4–6); (**B**) ROS production was measured by DCFDA fluorescence assay. Results are expressed as the mean fold alteration above or below the control (25 mM Glu) (±S.D.) (*n =* 4–6); (**C**) The protein levels of p-ERK, ERK, p-GSK3β, GSK3β, and GAPDH were measured by Western blot analysis; (**D**) The protein levels of Bax, Bcl-2, PARP, and GAPDH were measured by Western blot analysis. The representative blots are shown and densitometry was used to quantify the protein levels. Each bar graph shows the mean fold decrease below the control (25 mM Glu) (±S.D.) (*n =* 3). Differences were considered statistically significant at * *p* < 0.05, ** *p* < 0.01, and *** *p* < 0.001 vs. 25 mM Glu and # *p* < 0.5, ## *p* < 0.01, and ### *p* < 0.001 vs. 1 mM Glu.

A previous study had mentioned that low glucose enhanced the cytotoxicity of metformin in various cancer cell lines [25]. We tested metformin at various doses (0.6, 1.2, or 2 mM) in SH-SY5Y neuroblastoma cells (Figure 3A). Interestingly, metformin further decreased the viability of cells cultured at 1 mM glucose in a dose-dependent manner, whereas metformin did not affect the cell viability in normal glucose condition. These data suggest that the glucose level determines metformin-induced cell death in SH-SY5Y cells. Glucose deficiency-induced cell death is associated with ROS production (Figure 2). Therefore, we next investigated whether metformin-induced cell death at 1 mM glucose is related to ROS production. Increased dose of metformin was found to further increase the ROS production in a glucose deficient condition, while metformin had no effect on ROS production in a normal glucose condition (Figure 3B), suggesting the involvement of ROS production in low glucose level-dependent cytotoxicity of metformin. In a glucose deficient condition, metformin treatment significantly decreased the phosphorylation of ERK and GSK3β in a dose-dependent manner (Figure 3C). In addition, the protein expression of Bax was significantly increased and the protein expression of Bcl-2 was decreased by metformin treatment during glucose deficiency. Metformin treatment also increased PARP cleavage in a dose-dependent manner (Figure 3D).

### 2.4. D-BHB Attenuates Glucose Deficiency-Induced Cytotoxicity and ROS Production in SH-SY5Y Cells

**Figure 4:**
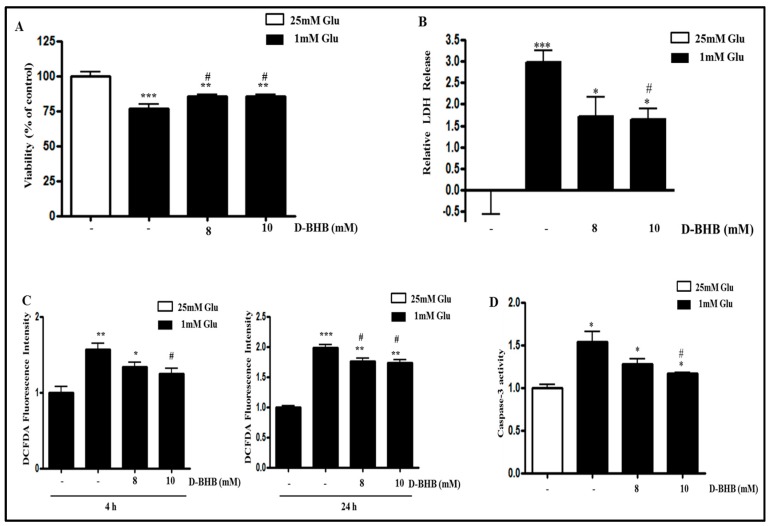
d-β-hydroxybutyrate (D-BHB) attenuates glucose deficiency-induced cytotoxicity and ROS production in SH-SY5Y cells. SH-SY5Y cells were pre-incubated with 1 mM glucose for 4 h, and then they were treated with D-BHB (8 or 10 mM) for 24 h. Control cells were incubated with 25 mM glucose. (**A**) The cell viability was measured by MTT assay; (**B**) The cell culture medium samples were taken and cytotoxicity was determined using lactate dehydrogenase (LDH) assay. Each bar represents the mean percentage alteration above or below the control (±S.D.) (*n =* 3); (**C**) ROS production was measured by DCFDA fluorescence assay; (**D**) Caspase-3 activity was measured using caspase-3 activity assay kit. Each bar represents the mean fold alteration above or below the control (±S.D.) (*n =* 3). Differences were considered statistically significant at * *p* < 0.05, ** *p* < 0.01, and *** *p* < 0.001 vs. 25 mM Glu and # *p* < 0.5 vs. 1 mM Glu.

We next evaluated the effect of D-BHB on glucose deficiency-induced cell death. MTT and lactate dehydrogenase (LDH) assays showed that the cytotoxicity induced by low glucose was partially but significantly reversed by treatment with D-BHB (Figure 4A,B). We next examined the effect of D-BHB on ROS production induced by glucose deficiency in SH-SY5Y cells. D-BHB treatment restored the glucose deficiency-increased ROS level (Figure 4C) and caspase-3 activity (Figure 4D), suggesting that the protective effect of D-BHB in glucose deficiency occurs through reduction of ROS production.

### 2.5. D-BHB Reverses Glucose Deficiency-Induced Reduction in the Phosphorylation of ERK and GSK3β in SH-SY5Y Cells

**Figure 5:**
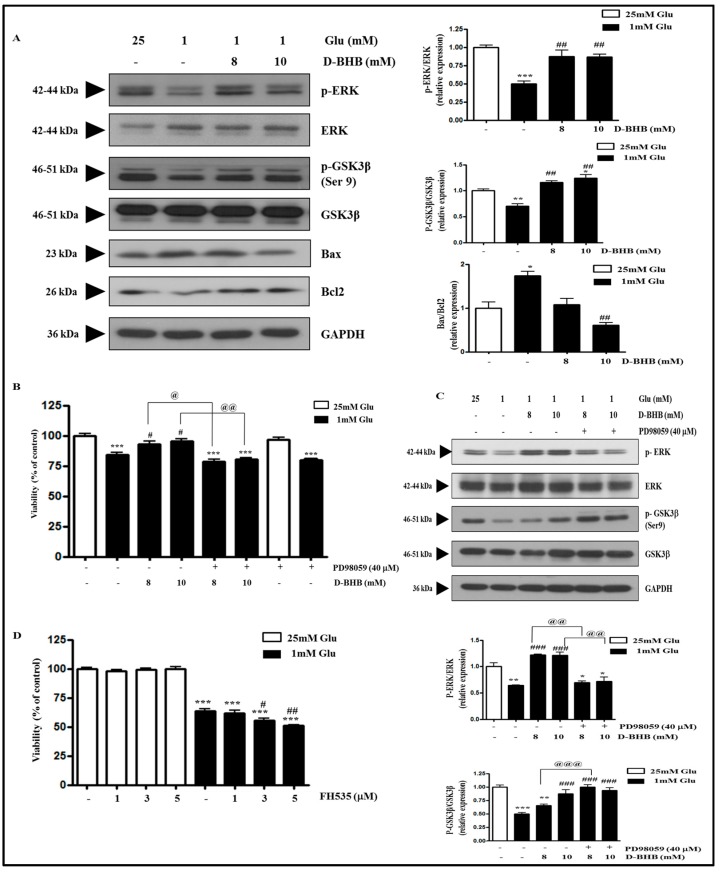
D-BHB reverses glucose deficiency-induced reduction in phosphorylation of ERK and GSK3β and PD98059 attenuates D-BHB-induced increase in cell viability in SH-SY5Y cells. (**A**) SH-SY5Y cells were pre- incubated with 1 mM glucose for 4 h, and then they were treated with D-BHB (8 or 10 mM) for 4 h. Control cells were treated with 25 mM glucose. The cells were lysed, and the expression of p-ERK, ERK, p-GSK3β, GSK3β, Bax, Bcl2, and GAPDH was measured by Western blot analysis. The representative blots are shown and densitometry was used to quantify the protein levels. Results are expressed as the mean fold alteration above or below the control (±S.D.) (*n* = 3); (**B**) SH-SY5Y cells were treated with D-BHB (8 or 10 mM) with or without 40 μM PD98059 for 24 h in 1 mM glucose media. Cell viability was measured by MTT assay. Each bar represents the mean percentage alteration above or below the control (±S.D.) (*n* = 3); (**C**) The expression of p-ERK, ERK, p-GSK3β, GSK3β, and GAPDH protein was determined by Western blot analysis. Densitometry was used to quantify bands. The representative blots are shown and densitometry was used to quantify the protein levels. The results are expressed as the mean fold alteration above or below the control (±S.D.) (*n =* 3); (**D**) SH-SY5Y cells were treated with 1, 3, or 5 μM FH535 for 24 h in 25 or 1 mM glucose media. The cell viability was measured by MTT assay. Results are expressed as the mean percentage decrease below the control (25 mM) (±S.D.) (*n =* 6). Differences were considered statistically significant at * *p* < 0.05, ** *p* < 0.01 and *** *p* < 0.001 vs. 25 mM Glu, # *p* < 0.5, ## *p* < 0.01, and ### *p* <0.001 vs. 1 mM Glu, and @ *p* < 0.5, @@ *p* < 0.01, and @@@ *p* < 0.001 vs. D-BHB.

Increased ROS production in glucose deficiency mediated decreased phosphorylation of ERK and GSK3β (Figure 2). Since D-BHB decreased ROS production, we next examined whether D-BHB restored the decreased phosphorylation of ERK and GSK3β induced by glucose deficiency. The phosphorylation of ERK and GSK3β was decreased by 1 mM glucose, which was reversed by D-BHB treatment (Figure 5A). In addition, D-BHB treatment attenuated the Bax/Bcl2 ratio which was increased by 1 mM glucose (Figure 5A). We examined whether the D-BHB-induced protective effect against glucose deficiency is attributable to increased phosphorylation of ERK and GSK3β. Increased cell viability induced by D-BHB co-treatment was suppressed by treatment with ERK inhibitor PD98059 (Figure 5B). Interestingly, ERK inhibition in D-BHB-induced protection during glucose deficiency did not decrease GSK3β phosphorylation, but increased it (Figure 5C). We next tested whether D-BHB-induced cell survival was mediated by the GSK3β signaling pathway. Increased phosphorylation of GSK3β at Serine 9 site induced by Akt leads to GSK3β inactivation [35]. GSK3β inhibitor FH535 further decreased cell viability in a glucose deficient condition, whereas it did not alter the cell viability in normal glucose condition (Figure 5D). These data suggest that glucose deficiency-induced cell death might not be mediated by activation of the GSK3β signaling pathway.

### 2.6. Metformin and D-BHB Affect Low Glucose-Induced Cell Death in Primary Neuronal Cells

**Figure 6:**
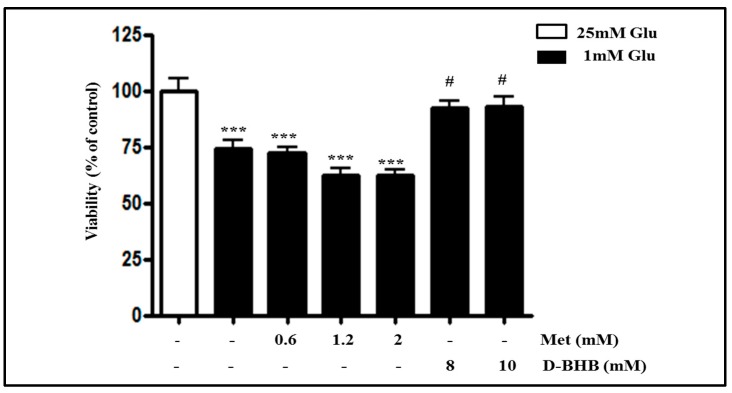
D-BHB attenuates glucose deficiency-induced cytotoxicity in primary neuronal cells. Primary neuronal cells were pre-incubated with 1 mM glucose for 4 h, and then they were treated with metformin (0.6, 1.2, or 2 mM) or D-BHB (8 or 10 mM) for 24 h. Control cells were incubated with 25 mM glucose. The cell viability was measured by MTT assay. Results are expressed as the mean percentage alteration above or below the control (±S.D.) (*n =* 6). Differences were considered statistically significant at *** *p* < 0.001 vs. 25 mM Glu, # *p* < 0.5 vs. 1 mM Glu.

We finally examined the effects of metformin and D-BHB in glucose deficiency using primary neuronal cells. As shown in Figure 6, cell viability in a low glucose condition was further reduced, but was not significantly decreased by metformin treatment, and it was partially restored by D-BHB treatment. These results are in agreement with our results in SH-SY5Y cells, suggesting the therapeutic potential of D-BHB in neurons in the brain.

## 3. Discussion

Hypoglycemia is an important acute complication in diabetes mellitus patients treated with insulin or sulfonylurea, which can cause severe brain damage. There are a number of ongoing studies to determine the mechanism of hypoglycemia-induced brain damage and the therapeutics that ameliorate the damage. In this study, we attempted to elucidate the molecular mechanism underlying hypoglycemia-induced neuronal cytotoxicity in vitro. For mimicking severe clinical hypoglycemia, 1 mM glucose-containing media was used instead of glucose deprivation. We also demonstrated that 25 mM glucose media used for general cell culture is not different from 5 mM glucose media, a normal blood glucose level of humans in the aspect of cell viability and the regulation of the major signaling molecules associated with the glucose deficiency. In addition, we also examined how D-BHB and metformin affected cell viability in a 1 mM glucose condition. We demonstrate that hypoglycemia-induced cell death is mediated by ERK inactivation through ROS production, which is reversed by D-BHB treatment, and aggravated by metformin treatment.

One of the most important findings of this study is that ERK is associated with glucose deficiency-induced cell death. ERK activation is associated with cell survival, proliferation, and differentiation in response to mitogens and cell survival factors [36,37]. However, it was recently reported that ERK activation also contributes to cell death in some cells and organs under certain conditions. Glutamate induced persistent ERK activation, and U0126, an inhibitor of ERK-activating kinase MEK1/2, attenuated glutamate-induced cytotoxicity in HT22 cells and primary cortical neurons [38]. In addition, ERK was activated in response to MPP^+^ in SH-SY5Y cells [39], amyloid in hippocampal neurons [40], or hydrogen peroxide in oligodendrocytes [41]. In this study, 1 mM glucose decreased cell viability and ERK phosphorylation, which were reversed by the treatment with NAC and D-BHB. Furthermore, the ERK inhibitor PD98059 suppressed D-BHB-induced increase in cell viability under glucose deficiency, suggesting a critical role of ERK activation in the protective effect of D-BHB against glucose deficiency. Therefore, the role of ERK activation in cell death or survival might depend on the insult and its intracellular signaling pathway. Further study is needed to clarify this issue.

On the contrary, it is unlikely that GSK3β inhibition has a protective effect against glucose deficiency-induced cytotoxicity. GSK3β was first recognized as a key protein kinase involved in glycogen metabolism, but it is now known to modulate various biological events including proliferation [12], differentiation [28], migration [26], glucose regulation [29], and apoptosis [30]. GSK3β is abundant in brain tissues, especially neurons, and it plays an important role in neuronal signaling [35,42]. In particular, GSK3β inhibition through PI3K/Akt phosphorylation is neuroprotective against ischemic injury [43]. In this study, glucose deficiency increased the GSK3β activity through reducing its inhibitory phosphorylation, which was reversed by treatment with the ROS inhibitor NAC. However, GSK3β inhibitor FH535 further decreased the viability of 1 mM glucose-treated cells, while it did not alter the cell viability of 25 mM glucose-treated cells. Furthermore, the inhibitory phosphorylation of GSK3β was increased when the ERK inhibitor reversed the D-BHB-induced protection against glucose deficiency. In this regard, glucose deficiency-induced cytotoxicity might not be mediated by GSK3β activation.

Cell culture at 1 mM glucose decreased the cell viability from 2 h onwards, but it was significantly decreased from 8 to 24 h. While glucose deficiency gradually increased ROS production, ATP content was slightly increased for up to 4 h of glucose deficiency, but it was significantly decreased from 12 h onwards (Figure 1B). However, the ATP level was more than 50% even at 24 h after glucose deficiency. Consistently, the intracellular ATP level was reported to be increased 3 h after glucose deprivation in PC12 cells [3]. ATP and other intermediates of the biosynthetic process are generated from glucose metabolism. There might be other energy sources, such as amino acids, in culture media which could be used to generate ATP at the beginning. Although the intracellular ATP level was transiently increased and then it was decreased during glucose deficiency, cell viability was continuously decreased from 2 h after glucose deficiency (Figure 1A), suggesting that glucose deficiency-induced cell death at early points in time is not attributable to ATP deficiency, which leads to necrotic cell death [44]. The increased PARP cleavage, caspase-3 activity, and Bax/Bcl-2 ratio also revealed that glucose deficiency induced apoptotic cell death.

This study clearly demonstrates that D-BHB has a protective effect against glucose deficiency-induced cytotoxicity in both SH-SY5Y cells and primary neuronal cells. In a previous report, D-BHB increased ATP production as an alternative substrate for energy and decreased ROS levels in glucose-deprived primary cortical neurons [12]. Ketone bodies were reported to have a scavenging action against the hydroxyl radical (OH) and to attenuate glycolysis inhibition-induced ROS production [8]. In accordance with this report, D-BHB treatment attenuated ROS production under 1 mM glucose, which was accompanied by decreased caspase-3 activity. Therefore, D-BHB has therapeutic potential not only in glucose deprivation but also in low glucose conditions. Furthermore, the protective effect of D-BHB is mediated by ERK activation.

The anticancer effect of metformin has been reported to result from activation of AMPK and inhibition of the mTOR signaling pathway [45]. In SH-SY5Y cells, metformin has been demonstrated to have an anticancer effect [21,22] and a neuroprotective effect in response to MPP^+^ [23] or α-synuclein [24]. Our data showed that metformin did not alter cell viability under 25 mM glucose, whereas it further increased cell death under 1 mM glucose. In this regard, metformin-induced cytotoxicity is determined by the glucose level, which might account for its converse effects between anticancer activity and neuroprotection. Interestingly, metformin resulted in ROS production depending on the glucose level. Although metformin did not alter ROS production under 25 mM glucose, it significantly increased ROS production under 1 mM glucose. ROS formation was accompanied by a decrease in the phosphorylation of ERK and GSK3β and by an increase in PARP cleavage and Bax/Bcl-2 ratio. In breast cancers, metformin treatment under 25 mM glucose showed no alteration in ATP levels, but metformin treatment under 2.5 mM glucose significantly decreased ATP levels [25]. Metformin enhances glycolytic metabolism and blocks mitochondrial oxidative metabolism. Therefore, metformin treatment with limited fuel source might decrease ATP production and increase ROS production.

In this study, we examined the molecular mechanism in hypoglycemia-induced neuronal cell death. We observed that 1 mM glucose increased ROS production and decreased the phosphorylation of ERK and GSK3β in SH-SY5Y cells. Metformin intensified 1 mM glucose-induced cell death, ROS production, and the decreased phosphorylation of ERK and GSK3β, while D-BHB reversed 1 mM glucose-induced cell death, attenuated ROS production, and restored the decrease in phosphorylation of ERK and GSK3β. D-BHB-induced protective effect in glucose deficiency was mediated by ERK activation, but not by GSK3β inhibition. Our study supports and extends previous observations of the neuroprotective effect of D-BHB and the anticancer effect of metformin under a glucose deficient condition. Furthermore, we demonstrate for the first time that ERK plays an important role in the effect of D-BHB in a glucose deficient condition.

## 4. Materials and Methods

### 4.1. Materials

Dulbecco’s Modified Eagle’s Medium (DMEM) with glucose and without glucose and all medium additives were obtained from Gibco BRL (Grand Island, NE, USA). Methylthiazolyldiphenyl-tetrazolium bromide (MTT), glucose, metformin, *N*-acetyl-l-cysteine (NAC), FH535, and 2-7-Dichlorodihydrofluorescein diacetate (DCFDA) fluorescence kit were purchased from Sigma Aldrich (St. Louis, MI, USA). PD98059 was purchased from Calbiochem (La Jolla, CA, USA). LDH, Caspase-3 activity assay kit and ATP measurement kits were purchased from BioVision (Milpitas, CA, USA). The antibodies specific for ERK, p-ERK, GSK3β, p-GSK3β, and PARP were purchased from Cell Signaling Technology (Danvers, MA, USA). The antibodies specific for Bax and Bcl-2 were purchased from Santa Cruz Biotechnology (Santa Cruz, CA, USA), and the antibody specific for GAPDH was purchased from Thermo Fisher (Rockford, IL, USA).

### 4.2. Cell Culture

Human neuroblastoma SH-SY5Y cells were cultured in Dulbecco’s modified eagle medium (DMEM) supplemented with 10% fetal bovine serum (FBS), 4.5 g/L d-glucose (25 mM), 2 mM glutamine, penicillin (100 U/mL), and streptomycin (100 μg/mL). Cells were maintained at 37 °C in a humidified atmosphere of 5% CO_2_. Primary neuronal cells were prepared from ICR mouse (embryonic day 17). ICR mouse was housed in a 12 h/12 h light-dark cycle with food and water provided ad libitum. All animal experimental protocols were approved by Institutional Animal Care and Use Committee of Wonkwang University, South Korea (Approval number: WKU13-49, November 2013) and all experiments were performed in accordance with the guidelines of the National Institute of Toxicological Research of the Korea Food and Drug administration. After isolation and removal of the embryo meninges, the cerebral cortex was transferred to Hank’s solution. After trypsinization for 30 min, the cells were suspended in 5% FBS-DMEM media and cultured on poly-d-lysine coated dishes in 5% CO_2_ atmosphere at 37 °C. After 5 h, the media were replaced with Neurobasal media supplemented with 0.4 mM glutamine and B-27. The cells were treated with 0.5 μM cytarabine to inhibit the growth of astrocytes 24 h later and reagent treatments were performed 24 h afterwards.

### 4.3. MTT Viability Assay

Cell viability was calculated by the reduction of MTT. SH-SY5Y cells were seeded at 1 × 10^5^ cells/well on a 96-well plate and treated with the 100 μL media containing 1, 5, or 25 mM glucose for the indicated time. In some experiments, the cells were treated with metformin, D-BHB, or a vehicle in the media containing 1 or 25 mM glucose for the indicated time. In some experiments, the cells were co-treated with ERK inhibitor PD98059, GSK3β inhibitor FH535, or ROS inhibitor NAC. After treatment, 100 μL of 1 mg/mL MTT was added for 2 h. The formazan was solubilized by 100 μL of dimethyl sulfoxide (DMSO) (Sigma Aldrich) for 20 min and the absorbance was measured by a multi plate reader at 540 nm (Bio-Rad, Hercules, CA, USA). Results are expressed as percentage alteration above or below the control.

### 4.4. LDH Cytotoxicity Assay

The cytotoxic activity was evaluated by measuring the key enzyme, lactate dehydrogenase (LDH). Ten minutes prior to assay, the cells were incubated with 1% (Volume/Volume) Triton X-100 in culture medium to acquire the maximal LDH release as the positive control. The cells were treated with 1 mM glucose with or without various doses of metformin for the indicated time. At the end of treatment, culture media were collected and centrifuged, and the supernatant was carefully transferred to a 96-well plate. The activity of LDH in the supernatant was determined using a LDH cytotoxicity detection kit according to the manufacturer’s instructions. The results were expressed as percentage of the maximum amount of LDH released from samples that had been treated with 1% Triton X-100. LDH release into media (%) was calculated using the following equation:

$$\mathrm{LDH}\;\mathrm{release}\;\left(\%\right)=\;\frac{\mathrm{OD}\;\mathrm{of}\;\mathrm{the}\;\mathrm{treated}\;\mathrm{well}\;-\;\mathrm{OD}\;\mathrm{of}\;\mathrm{the}\;\mathrm{low}\;\mathrm{control}\;}{\mathrm{OD}\;\mathrm{of}\;\mathrm{the}\;\mathrm{high}\;\mathrm{control}\;-\;\mathrm{OD}\;\mathrm{of}\;\mathrm{the}\;\mathrm{low}\;\mathrm{control}}\times 100\%$$

### 4.5. ATP Measurement Assay

ATP measurement was performed according to the manufacturer’s instructions. SH-SY5Y cells were lysed with a lysis buffer, followed by centrifugation at 12,000× *g* for 5 min at 4 °C. Then, 100 μL of ATP assay buffer was added to the pellet in each tube and 20 μL of ice cold PCA and 4 μL of ice-cold Neutralizing solution were added in each tube. Then, the samples were ready to be assayed and the absorbance was measured by a multi-plate reader at 570 nm.

### 4.6. DCFDA Fluorescence Assay

The generation of ROS was measured using DCFDA Fluorescence kit according to the manufacturer’s instructions (Sigma Aldrich). SH-SY5Y cells were seeded at 1 × 10^5^ cells/well on a 96-well plate and treated with the indicated reagents. In the positive control, 500 μM of H_2_O_2_ was added 45 min prior to completion of treatments. Diluted DCFDA was added for 30 min, and detected using fluorescence spectroscopy with excitation at 485 nm and emission at 535 nm wavelengths. For fluorescence microscopic images, SH-SY5Y cells were seeded on 6-well plates. Cells were treated with the fluorescent dye, DCFDA, and they were kept in an incubator for 30 min. Then, the cells were washed with PBS to remove the excess dye. The images were taken using a fluorescence microscope.

### 4.7. Caspase-3 Activity Assay

The apoptosis was determined by the level of activation of caspase-3 which was measured by using Caspase-3/CPP32 colorimetric assay kit (Biovision). The SH-SY5Y cells were seeded at 2 × 10^6^ cells per well in 6-well plate and after 24 h incubation, treated with 25 or 1 mM glucose containing media for indicated time or with media containing 1, 5, or 25 mM glucose for 24 h. Then, caspase-3 activity was measured as per the procedure given by the manufacturer.

### 4.8. Western Blot Analysis

SH-SY5Y cells were treated with the indicated reagents, and lysed with radioimmunoprecipitation assay (RIPA) buffer supplemented with a protease inhibitor and proteasome cocktail (Roche-Life Science, Mannheim, Germany). Protein concentrations in cell lysates were determined using bicinchoninic acid (BCA) protein assay kit (Thermo Scientific, Illinois, Rockford, AL, USA). The protein lysates were subjected to sodium dodecyl sulfate polyacrylamide gel electrophoresis (SDS-PAGE) and transferred to polyvinylidinedifluoride (PVDF) membranes (Millipore, Badford, MA, USA). After blocking with 5% skim milk in Tris-buffered saline (TBS) buffer, the membranes were incubated with antibodies specific for p-ERK, ERK, p-GSK3β, GSK3β, PARP, Bax, Bcl-2, and GAPDH (dilution 1:1000) overnight at 4 °C. The membranes were then incubated with the appropriate secondary antibody coupled to horseradish peroxidase (HRP) (dilution 1:3000) (Invitrogen, Carlsbad, CA, USA) for 1 h. The blots were then developed in a chemiluminescent mixture (Thermo Scientific), and exposed X-ray film (Fujifilm, Minato, Tokyo, Japan). The relative intensities of specific protein bands were determined by densitometry using ImageJ computer-assisted image analysis system.

### 4.9. Statistical Analysis

All experiments were performed at least three times using independent datasets with identical results. All results are expressed as mean ± standard deviation (S.D.), and the presented figures are representative of a series of experiments. Statistical significance of the difference was determined using one-way analysis of variance (one-way ANOVA). Tukey test was used for comparing the paired sets of data, and Dunnett test was used for multiple sets of data. A value of *p* < 0.05 was considered statistically significant.
